# Designing a Low-Cost System to Monitor the Structural Behavior of Street Lighting Poles in Smart Cities

**DOI:** 10.3390/s23156993

**Published:** 2023-08-07

**Authors:** Antonino Quattrocchi, Francesco Martella, Valeria Lukaj, Rocco De Leo, Massimo Villari, Roberto Montanini

**Affiliations:** 1 Department of Engineering, University of Messina, C.da di Dio, 98166 Messina, Italy; francesco.martella@unime.it (F.M.); valeria.lukaj@unime.it (V.L.); rocco.deleo@studenti.unime.it (R.D.L.); roberto.montanini@unime.it (R.M.); 2 Department of Mathematics, Computer Science, Physics and Earth Science (MIFT), University of Messina, Viale Ferdinando Stagno d’Alcontres 31, 98166 Messina, Italy; massimo.villari@unime.it

**Keywords:** Structural Health Monitoring (SHM), urban and suburban frameworks, real-time visualization, Internet of Things (IoT), Edge/Cloud systems, metrological performance, uncertainty budget, meteorological parameters, acceleration, tilt

## Abstract

The structural collapse of a street lighting pole represents an aspect that is often underestimated and unpredictable, but of relevant importance for the safety of people and things. These events are complex to evaluate since several sources of damage are involved. In addition, traditional inspection methods are ineffective, do not correctly quantify the residual life of poles, and are inefficient, requiring enormous costs associated with the vastness of elements to be investigated. An advantageous alternative is to adopt a distributed type of Structural Health Monitoring (SHM) technique based on the Internet of Things (IoT). This paper proposes the design of a low-cost system, which is also easy to integrate in current infrastructures, for monitoring the structural behavior of street lighting poles in Smart Cities. At the same time, this device collects previous structural information and offers some secondary functionalities related to its application, such as meteorological information. Furthermore, this paper intends to lay the foundations for the development of a method that is able to avoid the collapse of the poles. Specifically, the implementation phase is described in the aspects concerning low-cost devices and sensors for data acquisition and transmission and the strategies of information technologies (ITs), such as Cloud/Edge approaches, for storing, processing and presenting the achieved measurements. Finally, an experimental evaluation of the metrological performance of the sensing features of this system is reported. The main results highlight that the employment of low-cost equipment and open-source software has a double implication. On one hand, they entail advantages such as limited costs and flexibility to accommodate the specific necessities of the interested user. On the other hand, the used sensors require an indispensable metrological evaluation of their performance due to encountered issues relating to calibration, reliability and uncertainty.

## 1. Introduction

Street lighting is an indispensable and crucial system in urban and suburban frameworks. Its main purpose is to prolong the activities of a city in the dark hours, illuminating places and streets traveled by pedestrians and vehicles. However, this involves significant costs; it requires about 13–14% of the annual production of global electricity [1]. In the context of Smart Cities, street lighting is not only reduced to an energy-intensive system to be optimized, but its infrastructure can be easily employed to understand and to improve vital urban parameters. This can be achieved not only by applying new-generation strategies, but also by intervening on current systems to make them innovative. The use of a sensor network and some specific communication technologies can transform street lighting poles, which are also approximately indicated as columns, into intelligent and multifunctional structures [2]. For example, it is possible to monitor environmental parameters [3], to manage vehicular traffic [4] or to implement the safety policies of a city [5,6]. These necessities have played prominent roles in society, and a large part of recent scientific and industrial efforts are directed in such a way.

A relevant aspect, which is especially underestimated in the less frequented and more peripheral areas of a city, is the decrease in structural safety over time. A more widespread, real-time and low-cost monitoring represents a possibility of considerable interest [7]. Although an electrical malfunction [8] is really frequent, the structural collapse of street lighting poles [9] is often unpredictable and particularly destructive. Traditionally, these supports were manufactured with wood [10] due to the abundant availability and low cost of such a material. However, since they consist of an organic material, they are therefore subject to a rapid deterioration, which is mainly caused by atmospheric agents. To cope with this adversity, they were replaced with concrete, cast iron or stainless-steel columns [11,12,13], which are more durable but also heavier, difficult to install and expensive. One applied alternative was structural steel [14]. Unfortunately, it is prone to corrosion and requires an anti-rust coating, which increases its maintenance costs. More recently, aluminum poles have been adopted [15], but fiber-reinforced polymers are currently preferred as manufacturing materials [16]. In fact, the latter are able to combine good mechanical properties, great durability and ease of installation. The poles are designed according to some specific regulations that take into consideration several factors, including their weight, the weight of their accessories, the acting forces of wind and snow, etc. [17,18]. In such a case, the main cause of degradation is chemical corrosion caused by atmospheric agents, pollutants, the composition of the installation soil and galvanic couplings, which are present in different urban contexts [19]. Specifically, the part of a structure that is most subject to this degenerative phenomenon is the one in correspondence with the joint with the ground, where there is the maximum static load. It is summarily hidden underground or concealed by protective sheaths and a concrete base. For this reason, the prediction of possible structural damage becomes extensively complex [20]. However, a structural collapse can also occur due to extreme causes, such as road accidents [21] and adverse weather conditions [22]. In any case, the collapse due to structural fatigue induced by normal atmospheric conditions is configured as primary and, above all, sudden [23].

Taking into account the previously described sources of damage, the residual life of a generic street lighting pole is a complex parameter to assess and is subject to various evaluation errors [24]. Typically, it is estimated by computing the corrosion rate and measuring the residual thickness of the structure [25,26,27] or by adopting a nondestructive method [28,29]. Few researchers have proposed different alternatives. For example, Ziolkowski et al. [30] analyzed the response of some artificial defects in poles by means of short-range ultrasonic guided wave technology and time–frequency decomposition and conceptualized this procedure for an executive application. Although such techniques remain valid locally and, above all, in specific cases, the large number of installed columns discourages their applicability on a massive scale due to the unsustainability of noncontact tests and enormous costs [31]. Currently, the most employed method remains manual or semi-automatic visual inspection unit by unit [32].

An attractive alternative is to perform an SHM (Structural Health Monitoring) strategy employing a dedicated system integrated in single street lighting poles and in accordance with the typical exigencies of a Smart City [33]. Currently, this remains a field of interest that is still limited both in the literature and on the market. Steinbauer et al. [34] have recently developed a method based on the determination of the natural frequency shift through environmental excitation from wind or traffic. Specifically, they employed a device consisting of a MEMS (Microelectromechanical System) accelerometer glued to the top of a pole. It was connected to a wireless network grid that was capable of sending the obtained measurements to a Cloud system and subsequently achieving the results from a prototype software developed in a Matlab/Simulink environment. Reverberi Enetec srl [35] introduced a triaxial accelerometer and a triaxial inclinometer in the nodes of its commercial remote control systems. The collected information is transferred via a proprietary Internet infrastructure to another proprietary remote management software for a subsequent analysis. Through these data, it is possible to monitor the oscillation and tilt of public lighting poles and, in the event of anomalous variations (collisions or structural failures), to send an automatic alert to an emergency response team. The main disadvantage of this system concerns its intrinsic commercial nature, which involves the exploitation of specific sensors and proprietary software and infrastructure.

The presented paper reports the design, the implementation and the experimental characterization of a low-cost system for monitoring the structural behavior of street lighting poles in Smart Cities. It consists of an acquisition and transmission device wired to a specific set of sensors and connected to an Internet network. The aim of this work is to introduce a multifunctional device that can be easily integrated on an existent urban and suburban framework. It has the ability to collect the previous structural information of the poles, to simultaneously measure meteorological aspects and to lay the foundations for the development of a method that is able to avoid the collapse of the same poles. The monitoring architecture is presented in the aspects concerning the software programming, the management and the visualization strategy of the acquired data and the sending of specific alerts to the control room. The described experimentation has been focused on the estimation of the metrological performance of the proposed system.

## 2. Materials and Methods

### 2.1. Low-Cost Monitoring System

The proposed system was a low-cost dedicated hardware consisting of an acquisition and transmission device wired to a set of sensors. It was inserted in a commercial case in plastic material with an IP 56 protection rating for outdoor use. To the latter, a circular support was screwed to its bottom to fix it to a street lighting pole.

The device was a single-board computer (Raspberry Pi 3 B+) supplied by an electrical grid via an on–off switch and equipped with two LEDs (Light-Emitting Diodes) in order to indicate its working status (green LED = powered; red LED = not powered). It was employed to acquire information, to locally store and to subsequently send the collected information via mobile connection. The sensors (Table 1) had the aim of identifying possible structural instabilities or failures of the pole due to dynamic human and environmental effects, and to simultaneously monitor some meteorological parameters. Specifically, the ambient temperature and ambient humidity were measured using a DHT22 sensor [36]. It was fixed on a thermally insulating support on one side of the case and protected by a Stevenson screen in ASA (Acrylonitrile Styrene Acrylate), which is also suitable for outdoor use and is UV (UltraViolet) resistant. The visible light intensity (wavelength 400–700 nm) of the solar radiation was evaluated using a GY-302 sensor [37], which was installed on the top of the case and covered by an opalized glass bulb. This sensor was capable of guaranteeing the transmission of solar rays and the insulation from atmospheric agents. The vibration and tilt of the pole, due to environmental causes and the passage of vehicles, were instead assessed using a GY-521 module equipped with an MPU-6050 sensor [38] placed inside the case integrally. All sensors were directly powered by the single-board computer. The GY-521 module was adopted by considering the data reported in [34] and the typical dynamic behavior of a street lighting pole. This aspect, which is well consolidated in the literature [39], is of considerable relevance as it depends on numerous intrinsic factors of the structure (e.g., material, geometry, and weight) and external agents (ambient deterioration and damage caused by third parties).

**Figure 1:**
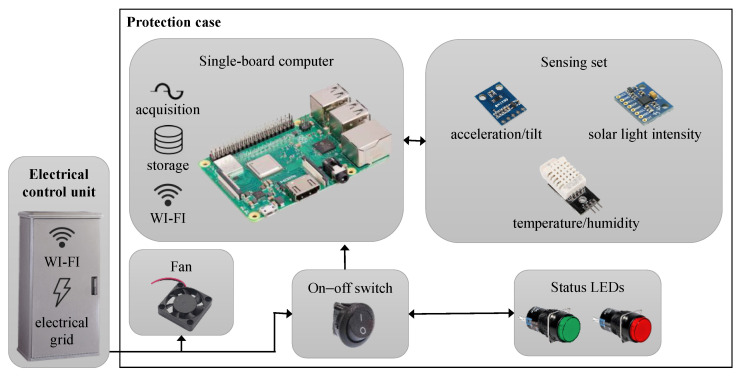
Representative schema of the electrical and electronical components of the monitoring system.

Finally, a small fan, supplied by a USB socket wired to an electrical grid, was installed to control the temperature and to expel the hot air through two vents on opposite sides of the case by the device. Figure 1 reports a representative schema of the electrical and electronic components.

**Figure 2:**
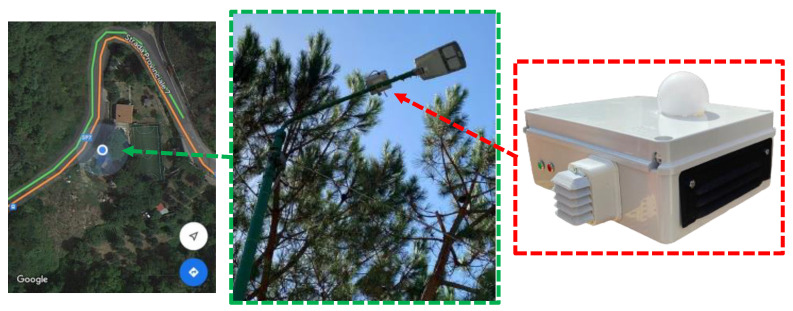
Installation and details of the monitoring system.

The proposed system had a volume of 190 × 140 × 70 mm^3^ and a weight of about 0.7 kg. It was installed on the top of a street lighting pole (Figure 2). The latter, about 10 m high, was chosen because it is located on a suburban road that is mainly frequented during the winter and summer holidays and is near a sports facility. Although the mechanical influence of the proposed system was not verified, the typical head of the pole has a volume and a weight 7–8 times superior. The structural resistance of the whole pole is ensured by its column [40]. In fact, although its accessories (e.g., head) are relevant to its mechanical behavior, their effect is often limited [41]. Moreover, a possible relamping must also be considered; in this case, the new lantern has a considerably lower weight than the old original one [42]. Specifically, the power supply was taken from an electrical grid to ensure there was a significant reduction in maintenance costs thanks to the unnecessary battery replacement. Instead, the communication to an Internet network was guaranteed via mobile 4G connectivity. In any case, the proposed solution can work, as it is associated with multiple devices with a single Internet entry point, which is also wired. The described situation constitutes the typical context of interest for the application presented in such a paper. Finally, taking advantage of the fact that it is based on a Raspberry computer and has a modular configuration, the proposed system can be easily integrated into the current lighting infrastructure and enriched with other functionalities. As an example, a further investigation procedure can be implemented [43].

### 2.2. Characterization Setup

**Figure 3:**
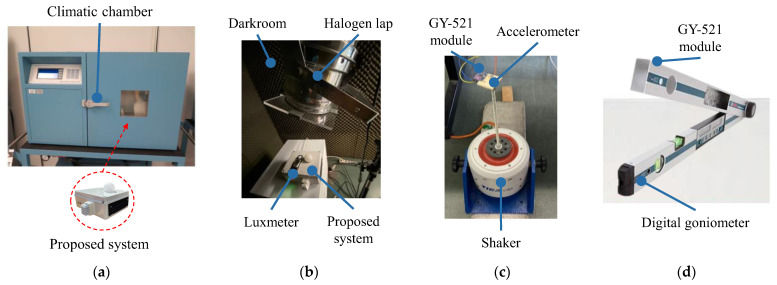
Characterization setup for the sensors of (**a**) ambient temperature and humidity, (**b**) visible light intensity, (**c**) acceleration and (**d**) tilt of the pole.

The proposed system w characterized by means of a different experimental setup (Table 2) for each specific function of the employed set of sensors. A climatic chamber [44] was employed to recreate the typical ambient conditions of the place where the proposed system was installed (Figure 3a). Specifically, as standard conditions, a constant Relative Humidity (RH) of 50% was chosen, and the temperature varied. Conversely, a temperature of 25 °C was set, and the RH changed. The visible light intensity was estimated in a darkroom (Figure 3b) at 50% RH and 25 °C as standard conditions. Inside, a halogen lamp with a maximum power of 1 kW, simulating the solar light, was used to project a controlled light beam onto the proposed system. The data were acquired and subsequently compared with those detected using a luxmeter [45]. An electrodynamic shaker (TIRA vib S 503, Schalkau, Germany [46]) supplied by a power amplifier (TIRA vib BAA 60, Schalkau, Germany [46]) and driven by a function generator (Agilent 33220A, Santa Clara, CA, USA [47]) was used to reproduce a sinusoidal dynamic stress (Figure 3c) at 50% RH and 25 °C. Specifically, a triaxial accelerometer [48] wired to an acquisition system (National Instrument cDAQ 3320, Austin, TX, USA [49]) was installed together with the GY-521 module of the proposed system on a rigid base (in aluminum with a thickness of 7 mm) directly fixed to the stinger (in steel with a diameter of 5 mm) of the same shaker. Finally, at 50% RH and 25 °C, a digital goniometer [50] was chosen to calibrate the GY-521 module, positioning the latter at a different tilt (Figure 3d), as reported in [51].

### 2.3. Cost Discussion

The adjective ‘low cost’ was adopted because the proposed system is based on low-cost equipment and open-source software. These factors intrinsically ensure that the system has a limited economic effort compared to commercial devices. Indeed, although it mainly depends on the volume of its production, the estimated cost of the complete proposed system is less than EUR 200. Specifically, the used sensors (DHT22, GY-302 and MPU-6050) represent less than 1/4 of the total cost, the electrical and mechanical small parts (cables, screws, circular support, protection case, etc.) and the auxiliary devices (fan, LED, etc.) represent just over 1/4 of the total cost and, finally, the single-board computer and its accessories represent about 1/2 of the total cost. Furthermore, as already reported, it should be considered that this system is not exclusive to the discussed application and can be implemented with further functions and other devices.

## 3. Results

### 3.1. Design of the IT Architecture

**Figure 4:**
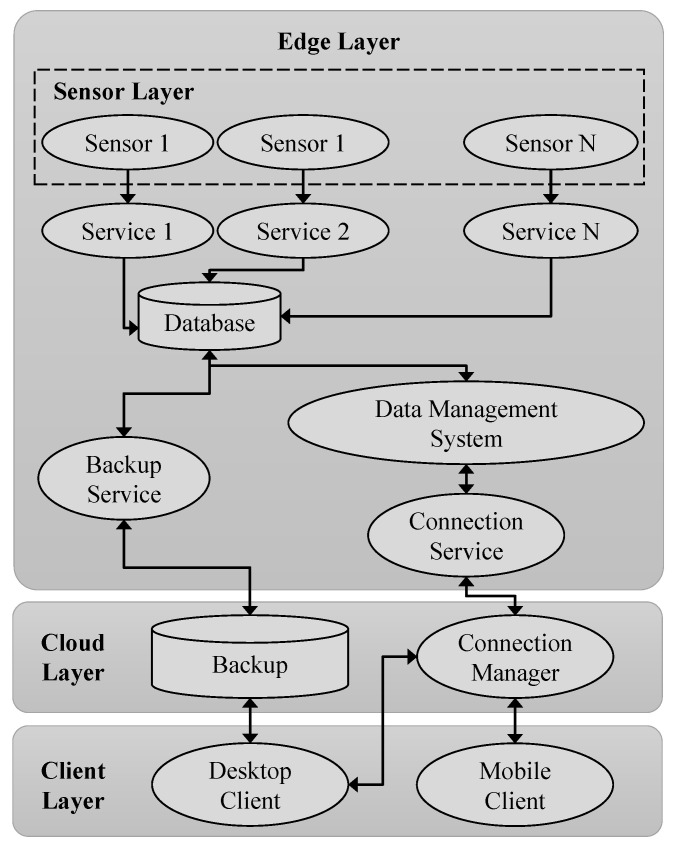
Schematic representation of the IT architecture.

The information technology (IT) architecture was based on a distributed Edge/IoT-Cloud system, which allowed the user to view, collect and manage the monitoring parameters, receiving dedicated notifications. Figure 4 depicts the architecture, which consisted of the following three main elements:

- Edge Layer. This defined the way to collect and to process the acquired data, to extract specific information and to monitor the results in real time. In addition, it was characterized by a higher level of complexity than the other elements of the monitoring architecture. In fact, it adopted a stratification and involved both parallel and confluent functions. In this work, the Edge structure consisted of the Sensor Layer, referring to the set of employed sensors (Sensor 1, … Sensor N). Their number and type depended on the GPIO (General Purpose Input Output) of the Edge device and on the necessary balance between their power consumption and the maximum electrical power provided by the device itself. A related service (Service 1, … Service N) was defined for each sensor. These were the software modules that read the data from the sensors and wrote them into a database using some dedicated drivers. For such a reason, they were strictly linked to the type of sensor and the DBMS (Database Management System) with which they interacted. Thus, the envisaged database allowed for the local storage of the acquired data using a Time Series DBMS. Two services simultaneously acted on the database. The first one was the Backup Service. It was a software module that periodically and automatically started a local backup of the database, transferred it to the Cloud and deleted it so as not to fill the device’s memory. The second one was the Data Management Service. This was a software module that allowed for the user to access to the database, to create customed dashboards for data visualization and to configure and send alert systems according to some specifications on the measured parameters. All of these last operations were guaranteed by the Connection Service, which consented to the user’s communication with the Edge device via the Cloud Layer. Such a type of service was chosen to control the user’s access to the device and to guarantee the security of the transferred data.
- Cloud Layer. This illustrated the elements that were necessary to remotely store and manage the collected data. Specifically, the Backup, i.e., a local database, was the component that periodically obtained updates from the Edge device via the Internet and kept a copy of these data. Moreover, it ran a software module (Connection Manager), which guaranteed the connection between the user and the Edge Layer. This connection was obtained via a VPN (Virtual Private Network) server. In this way, once the certificates were issued, the user could interrogate the Edge Layer by configuring their client.
- Client Layer. This described the apparatus and the software that was directly usable by the user to interact with the Edge device. The client application could be executed from both the desktop and mobile terminals, but with some substantially different purposes. A Desktop Client employed personal computers, laptops, etc., to have access to the dashboard and create a data backup via the browser and remote connection software. Instead, a Mobile Client with smartphones, tablets, etc., could contact the dashboard via a browser. However, with this limitation, such a method had the advantage of receiving alerts more immediately. For both cases, notifications could be received via email or messaging apps using specific bots.

### 3.2. Development of the IT Architecture

**Figure 5:**
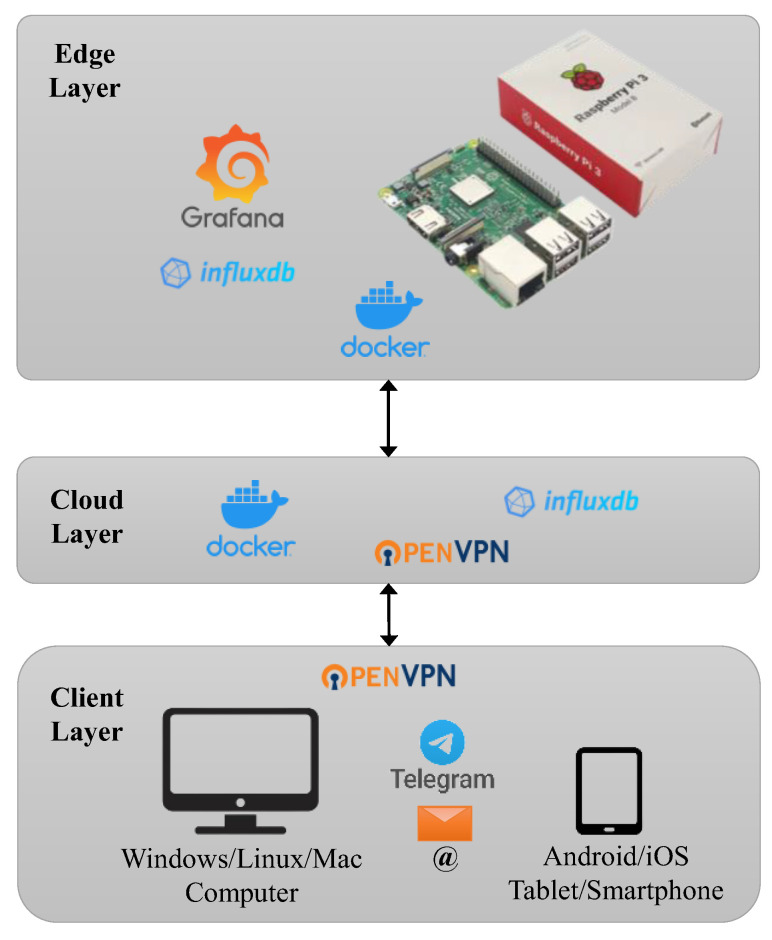
Schematic representation of the deployed scenario.

Figure 5 shows the main ITs employed for the development of each of the previously described layers (Figure 4).

The Edge Layer adopted the single-board computer (Raspberry Pi 3 B+) as an Edge device. It was wired to the employed set of sensors (i.e., Sensor Layer) via its GPIO and equipped with the native Raspian OS (Operating System). The collected data were saved on a database (i.e., Database), built with InfluxDB, by means of some dedicated scripts (i.e., Service 1, … Service N) written using the Python programming language, and managed using the Grafana web application (i.e., Database Management System). Furthermore, through the Linux OS commands, the automatic execution of the backup script (i.e., Backup Service), and the connection first to a WI-FI network and subsequently to a VPN network (i.e., Connection Service) for sending the previously saved data were set. InfluxDB, Grafana and the associated scripts were installed on the Edge device, using the container method of the Docker technology.

The Cloud Layer also exploited Docker for its services. Specifically, an InfluxDB instance performed a daily update of the backup (i.e., Backup) with all the data from the previous day, while a container instance of the server of the Open VPN software was used to issue certificates and allow for the user to connect to the Edge device (i.e., Connection Manager).

The Client Layer adopted desktop/laptop terminals with Windows/Mac/Linux OS (i.e., Desktop Client) or mobile ones with Android OS/iOS (i.e., Mobile Client), which accessed the Cloud Layer and the device via the OpenVPN and any browser. The respective features were performed via Grafana. Specifically, Grafana not only allowed for the access, the customization of the dashboard and the creation of specific data backups, but also allowed for the implementation through bots of dedicated alerts. These notifications, produced when the data reached defined thresholds, were sent via the messaging service of the Telegram app and also via e-mails, which can be received with any e-mail client.

### 3.3. Preliminary Field Test

**Figure 6:**
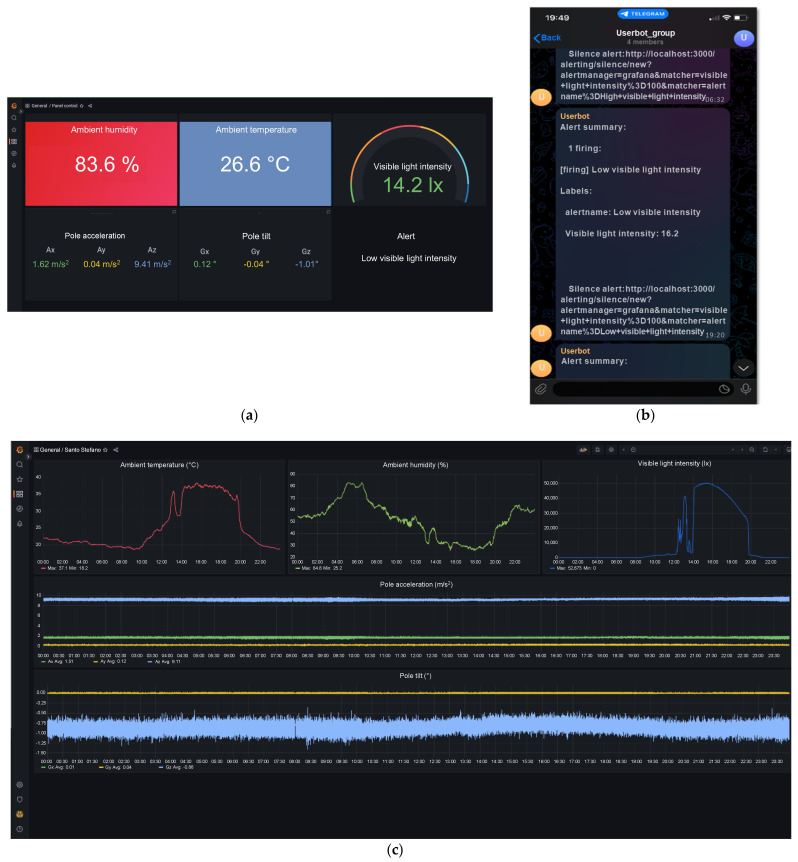
Typical interface, (**a**) indicators and (**c**) graphs customed by the uses on Grafana for desktop terminals, and typical alert for smartphone on Telegram app (**b**).

Figure 6 illustrates the typical interfaces of the deployed scenario during a preliminary field test.

The instantaneous values of the measured parameters and an alert are indicated in Figure 6a, while the related graphs are displayed in Figure 6b. This alert proved the status of the ‘low visible light intensity’ that was defined using a threshold of 100 lx. Instead, the time history of the acquired data was set at 24 h. As already stated in the previous section, according to the specific authorizations guaranteed by the suitable VPN certificates, the user is able to view a collected point or edit the different parts of the interface. As an example, the user can rescale the axes of each graph or choose to download a specific portion of the collected data. Finally, a screenshot of the display of a smartphone, on which an alert summary is provided by the Telegram app, is exhibited in Figure 6c.

A sampling frequency of 1 Hz was employed for the measurements of the ambient temperature, ambient humidity and visible light intensity. This was chosen because, reasonably, the monitored meteorological parameters vary slowly during the day. Instead, after considering exceptional events (as an example, strong windy conditions), the sampling frequency was set to 20 Hz for the acceleration and tilt of the pole. These values were only selected as preliminary ones.

The collected data show that the proposed system works in a suitable way, taking into account the environmental conditions in the place where the pole is installed. The trend of the ambient temperature is low during the night and the first part of the morning (22:00–9:30), increases during the second part of the morning (9:30–13:00), is high during the afternoon (13:00–19:30) and finally decreases during the evening (19:30–22:00). The ambient humidity has a more complicated drift, with a progressive rise starting from sunset and a reduction starting from sunrise. The visible light intensity displays a typical trend, but some considerations need to be made. The investigated pole is in the shade until 12:00 and thereafter is exposed to direct sunlight. Furthermore, some trees are close to the pole, and therefore, there are some important influences on the direct light. With regard to the acceleration of the pole, specific events are not clearly noted. This is due to the used time scale (24 h), which is unsuitable for short events, and due to the amplitude, which is capable of including the three components x, y and z. However, specific events can be identified during the early morning hours (8:00–8:30). These may be caused by the passage of some vehicles or by short gusts of wind. What is more interesting is the case of the tilt, where the angle along the vertical z direction (azure curve) highlights a possible and very slight relaxation of the pole during the hottest hours (12:00–19:30) and during the passage of some vehicles or short gusts of wind (8:00–8:30).

### 3.4. Metrological Performance

A metrological characterization was performed to investigate the possibility of implementing an accurate measurement system to monitor the structural behavior of street lighting poles in Smart Cities using low-cost devices.

**Figure 7:**
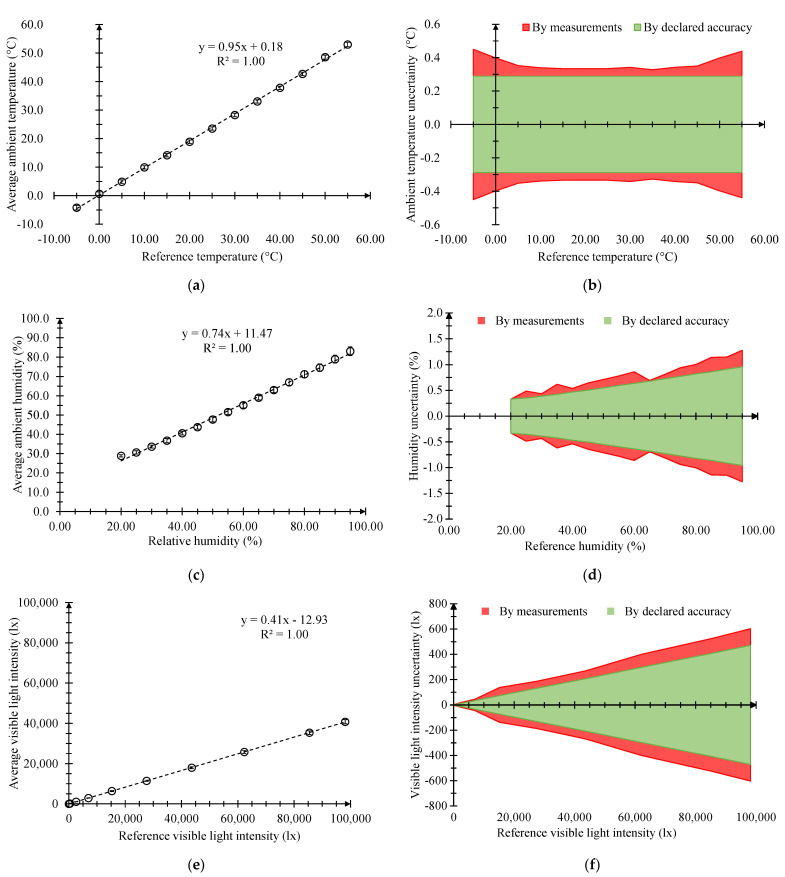
Calibration curves with bars of standard deviation and comparison between the trends of the uncertainties computed by the standard deviations of the collected measurements and by the declared accuracy for (**a**,**b**) ambient temperature at an RH of 50% and (**c**,**d**) ambient humidity at 25 °C, and for (**e**,**f**) visible light intensity at 50% RH and 25 °C of the proposed system.

Figure 7 reports the calibration curves and the comparison between the trends of the uncertainties, computed by the standard deviation of the collected measurements and by the declared accuracy (Table 1) of the proposed system with reference to the ambient temperature, ambient humidity and visible light intensity.

For the DHT22 (Figure 7a,c) and GY-302 (Figure 7e) sensors, each point of the calibration curves was obtained by considering the average value of three different measurements. The latter were calculated by averaging the last 10 points that were acquired 300 s after reaching stationary conditions at a sampling frequency of 10 Hz. These procedures were employed to obtain the values that best represent the performance of the investigated sensors. In fact, an average value of the specific information should be preferred in order to avoid potential outliers. The uncertainty budget was carried out in accordance with [52]. Specifically, the uncertainty relating to the collected measurements was estimated by dividing the value of the punctual standard deviation by $\sqrt{n}$, where *n* is the number of repetitions of the same type of measurement (i.e., 3). Instead, the uncertainty related to the data stated by the manufacturer was calculated by considering a rectangular distribution and therefore dividing the declared accuracy by $\sqrt{3}$.

The three calibration curves had a good linearity, but the collected average measurements were generally inferior to the reference ones in terms of the amplitude. Specifically, the curves of the ambient temperature and ambient humidity presented a limited divergence from the linear fitting, even if the latter of the ambient humidity was worse at the beginning of the measurement range. Instead, the sensor for the visible light intensity exhibited two different issues. On one hand, the acquired values were averagely reduced by 68% compared to the reference ones; on the other hand, the linear range of the calibration curve extended well above the maximum of 65,535 lx, as stated by the manufacturer (Table 1), up to 100,000 lx. Putting into evidence the trends of the uncertainty estimated by the standard deviations of the collected measurements (Figure 7b,d,f), it can be seen that they were similar to those estimated by the declared accuracy. There was only an uncommon behavior in the case of the ambient temperature. In fact, the variation of the uncertainty, estimated by the standard deviations, remained sufficiently constant between 5 and 45 °C, while before and after this range, it decreased and then increased rapidly in an approximately linear way. Another observation should be made for their average values. The average values were higher than those obtained by the declared accuracy by approximately 26%, 24% and 42% for the ambient temperature, humidity and visible light intensity sensors, respectively.

**Figure 8:**
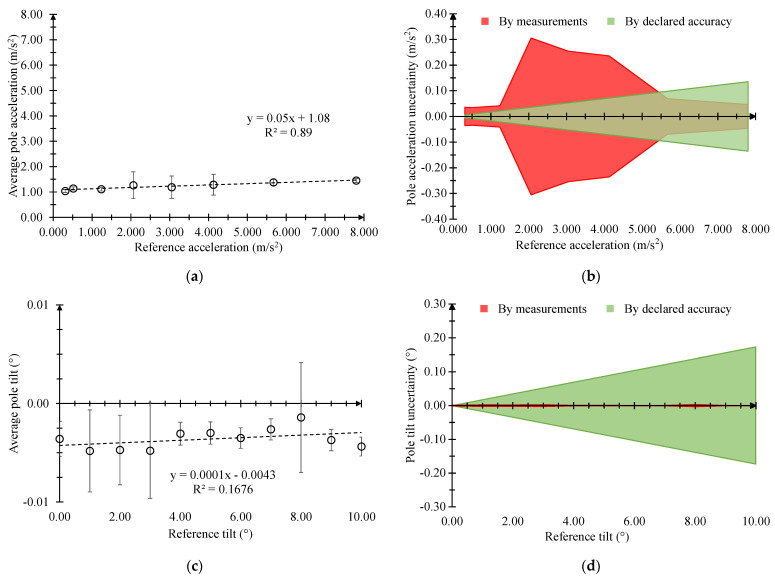
Calibration curves with bars of standard deviation and comparison between the trends of the uncertainties computed by the standard deviations of the collected measurements and by the declared accuracy for the MPU-6050 sensor in the GY-521 module for the vertical acceleration (**a**,**b**) and tilt (**c**,**d**) of the pole at 50% RH and 25 °C.

For the MPU-6050 sensor in the GY-521 module, each point of the calibration curves was also obtained by considering the average value of three different measurements. However, the latter were calculated by averaging the last 10 consecutive maximum points of the acceleration signal acquired 5 s after reaching stationary conditions at a sampling frequency of 100 Hz. The uncertainty budget was achieved in the same way as reported for the previous sensors. Nevertheless, a specific consideration must be made for the vertical acceleration and tilt of the pole (Figure 8).

In this case, the MPU-6050 sensor in the GY-521 module displayed an abnormal behavior; although the collected signals were stable, their amplitude was rather reduced, and their variability was remarkably high. According to a detailed analysis, the calibration curves (Figure 8a,c) exhibited a linear trend, but were also characterized by a fluctuating standard deviation (Figure 8b,d). Specifically, for the acceleration of the pole (Figure 8b), the uncertainty estimated by the standard deviations of the collected measurements was generally wider than that by the declared accuracy and above all did not have a defined trend, while for the tilt of the pole (Figure 8d), the first was significantly lower than the second one.

**Figure 9:**
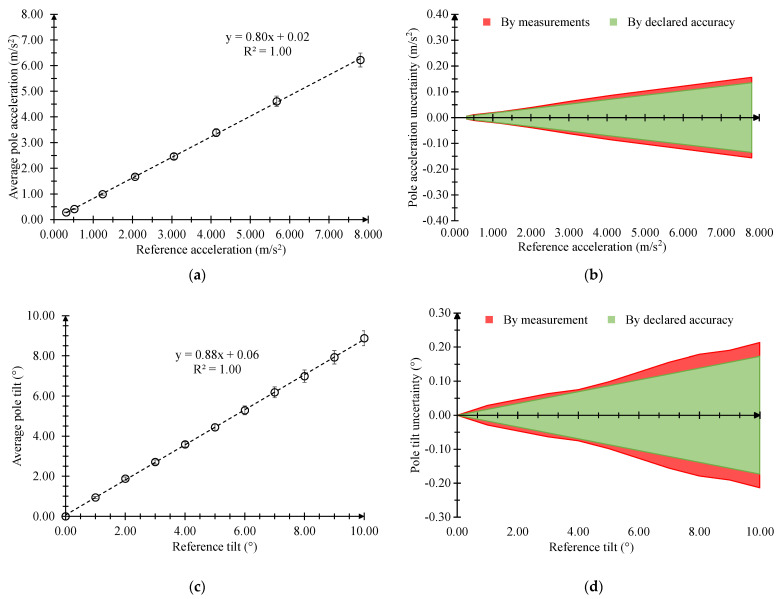
Calibration curves with bars of standard deviation and comparison between the trends of the uncertainties computed by the standard deviations of the collected measurements and by the declared accuracy for the MPU-6050 sensor in the GY-521_N2 module for the vertical acceleration (**a**,**b**) and tilt (**c**,**d**) of the pole at 50% RH and 25 °C.

According to the explained reasons, we proceeded by replacing the employed GY-521 module with a second one (GY-521_N2) from the same production batch and then re-evaluated its calibration curves, standard deviations and uncertainty components (Figure 9).

The calibration curves still confirmed a linear trend, but contrary to what was previously obtained in Figure 8a,c, the coefficients of the linear regression were significantly different, and the standard deviations were decisively limited and stable (Figure 9a,c). Comparing the uncertainty components, their trends (Figure 9b,d) appeared to be very changed from those reported in Figure 8b,d. In fact, the uncertainty estimated by the standard deviations of the collected measurements of the MPU-6050 sensor in the GY-521_N2 module was only slightly higher than the uncertainty estimated by the declared accuracy by approximately 18% and 27% for the pole acceleration and tilt sensor, respectively. 

## 4. Discussion and Conclusions

The work presented the design, implementation and experimental characterization of a low-cost system for monitoring the structural behavior of street lighting poles in Smart Cities. Specifically, it highlighted and investigated the benefits and drawbacks concerning economic aspects, including those of the field of information technology and of the science of measurements.

Contrary to many commercial systems, the proposed one was assembled by employing low-cost equipment (DHT22, GY-302, GY-521, etc.) and open-source software (Raspian OS, InfluxDB, Grafana, etc.). These materials and methods are the main reason to consider why the ‘low-cost’ adjective is appropriate. However, intrinsically, they ensure a significant cost reduction, considering the large number of required devices in such an application. Furthermore, the proposed system can be easily integrated into current infrastructures because they are characterized by a modular configuration according to the used single-board computer. Finally, the system shows a marked flexibility to accommodate the specific necessities of the concerned user. Indeed, the user is able to gain access to the collected information in real time and in a secure way via a VPN, to archive the measured data on the Edge device and also on the Cloud platform, to customize the software interface for the presentation of the results, to interrogate the same monitoring system from desktop and mobile terminals and, finally, to receive personalized notifications on the monitored parameters. Although the presented system was implemented to assess the acceleration and tilt of the pole, the specific set of sensors was selected with the intent of achieving a larger goal. According to the modularity feature of the proposed system and in order to lay the foundations for the development of a method that is able to avoid the collapse of the same poles, sensors for the ambient temperature, ambient humidity and visible light intensity were chosen. Specifically, the measurement of the visible light intensity, even if it is apparently not relevant for the prediction of the collapse of a pole, is typical of applications involving lighting infrastructures in Smart Cities.

On the other hand, after completing an experimental campaign to investigate their metrological performance, the low-cost devices demonstrated some disadvantages. First of all, although the calibration curves showed a good linear trend and a limited standard deviation in the measurement range, their amplitudes were generally lower than expected. Afterward, the estimated uncertainty by the standard deviations of the collected measurements was found to be much higher (min. 18%, max. 42%) than that of the accuracy stated by the manufacturer for each sensor. These two issues are certainly relevant as they involve both the underestimation of the measured parameters and a decrease in the reliability of the measurement itself. Further limitations highlighted during the experimental phase were found in the extended linearity of a sensor (that of the visible light intensity) beyond the declared measurement range and in the malfunctioning of another sensor (that of the acceleration and tilt). These behaviors can jeopardize the correct functioning of the whole measurement system. Therefore, the consistent and advantageous use of a system to monitor the structural behavior of street lighting poles based on low-cost devices, open-source software and Cloud/Edge strategies in order to prevent their collapse requires an indispensable metrological evaluation of the employed sensors. Finally, the proposed system can be employed as a starting point to ensure the achievement of a metrologically detailed structural monitoring on a large scale with low-cost equipment.

Future developments will be focused on the assessment of the operational reliability of the investigated system and on the definition of a methodology for predicting the collapse of street lighting poles. Then, a large-scale monitoring campaign will be carried out on both temporal and spatial terms. Furthermore, suitable alerts for the correct prediction of the collapse of such poles, as a consequence of dangerous human activity and environmental influences, will be identified.

## Figures and Tables

**Table 1:** Main features of the set of sensors.

Sensor ID	Measured Parameter	Range	Accuracy	Resolution	Max. Sampling Rate (Hz)	Working Temperature (°C)	Working Humidity (%)
DHT22	Ambient temperature	−40–80 °C	±0.5 °C	±0.1 °C	0.5	−40–80	0–100
DHT22	Ambient humidity	0–100%	±2%	0.1%	0.5	−40–80	0–100
GY-302	Visible light intensity	1–65,535 lx	±2%	0.1%	1	−40–85	0–95
MPU-6050 (in GY-521)	Pole acceleration	±8 g	±3%	±2%	1000	−40–85	0–95
MPU-6050 (in GY-521)	Pole tilt	±1000°/s	±3%	±2%	1000	−40–85	0–95

Accuracy and resolution are referred to the instantaneous measured value.

**Table 2:** Details of the characterization setup.

Equipment	Parameter	Model and Manufacturer	Testing Condition
Climatic chamber	Ambient temperature	Thunder Scientific 2500 lbuquerque, NM, USA [44]	50% RH
Climatic chamber	Ambient humidity	Thunder Scientific 2500 Albuquerque, NM, USA [44]	25 °C
Luxmeter	Visible light intensity	Testo 540 Settimo Milanese, Italy [45]	50% RH, 25 °C
Triaxial accelerometer	Pole acceleration	PCB Piezotronics 356A19 Depew, NY, USA [48]	50% RH, 25 °C
Digital goniometer	Pole tilt	BOSCH GAM 270 MFL Gerlingen, Germany [50]	50% RH, 25 °C

## Data Availability

Not applicable.
